# Seasonal Influenza Vaccination and Recommendation: The Difference between General Practitioners and Public Health Workers in China

**DOI:** 10.3390/vaccines8020265

**Published:** 2020-05-31

**Authors:** Hongguo Rong, Xiaozhen Lai, Xiaochen Ma, Zhiyuan Hou, Shunping Li, Rize Jing, Haijun Zhang, Zhibin Peng, Luzhao Feng, Hai Fang

**Affiliations:** 1 China Center for Health Development Studies, Peking University, Beijing 100083, China; hgrong@hsc.pku.edu.cn (H.R.); xma@hsc.pku.edu.cn (X.M.); 2 School of Public Health, Peking University, Beijing 100083, China; laixiaozhen@pku.edu.cn (X.L.); rzjing2015@hsc.pku.edu.cn (R.J.); zhanghj966@bjmu.edu.cn (H.Z.); 3 School of Public Health, Fudan University, Shanghai 200032, China; zyhou@fudan.edu.cn; 4 School of Health Care Management, Cheeloo College of Medicine, Shandong University, Jinan 250012, China; lishunping@sdu.edu.cn; 5 NHC Key Laboratory of Health Economics and Policy Research, Shandong University, Jinan 250012, China; 6 Division of Infectious Diseases, Chinese Center for Disease Control and Prevention, Beijing 102206, China; pengzb@chinacdc.cn; 7 Peking University Health Science Center—Chinese Center for Disease Control and Prevention Joint Center for Vaccine Economics, Beijing 100083, China; 8 Center for Infectious Disease Research and Policy, Peking University Institute for Global Health, Beijing 100083, China

**Keywords:** healthcare workers, influenza, vaccination, public health workers, general practitioners

## Abstract

Seasonal influenza vaccination for healthcare workers (HCWs) is critical to the protection of HCWs and their patients. This study examined whether the separation of public health workers and general practitioners could affect the influenza vaccine uptake and recommendation behaviors among HCWs in China. A survey was conducted from August to October 2019, and HCWs from 10 provinces in China were recruited. A self-administered and anonymous questionnaire was used to assess HCWs’ demographic information, knowledge, and attitudes toward influenza vaccination, as well as vaccine uptake and recommendation behaviors. The primary outcome was HCWs’ vaccination and recommendation status of seasonal influenza vaccine. Multivariate logistic regression models were used to identify the influence factors of influenza vaccine uptake and recommendation among HCWs. Of the 1159 HCWs in this study, 25.3% were vaccinated against influenza in the previous season. “No need to get vaccinated” was the primary reason for both unvaccinated public health workers and general practitioners. Multivariate logistic regression showed that public health workers were more likely to get vaccinated against influenza (OR = 2.20, 95% CI 1.59–3.05) and recommend influenza vaccination to children (OR = 2.10, 95% CI 1.57–2.80) and the elderly (OR = 1.69, 95% CI 1.26–2.25) than general practitioners. Besides, the knowledge and perceived risk of influenza can give rise to HCWs’ vaccination and recommendation behaviors, and HCWs who got vaccinated in the past year were more likely to recommend it to children and the elderly in their work. The influenza vaccine coverage and recommendation among HCWs are still relatively low in China, especially for general practitioners. Further efforts are needed to improve the knowledge and attitudes toward influenza and influenza vaccination among HCWs, and coherent training on immunization for both public health workers and general practitioners might be effective in the face of separated public health and clinical services in China.

## 1. Introduction

The World Health Organization (WHO) estimated that seasonal influenza may affect 5% to 15% of the world’s population each year, causing as many as 3–5 million severe cases and 290,000–650,000 deaths [1]. Accordingly, influenza places a huge burden on healthcare and social economy in most countries, including China [2]. At present, annual vaccination is the best way to prevent influenza infection [3].

Compared with the general population, healthcare workers (HCWs) are at higher risk of exposure to influenza virus, bringing potential threats for the occupational health of HCWs [4]. Meanwhile, HCWs may therefore represent an important vector for transmission to susceptible patients, including those at high risk for serious outcomes [5]. The technical guidance for influenza vaccination in China (2019–2020) clearly pointed out that HCWs are the priority population for influenza vaccination, together with children (aged between 6 months to 59 months), the elderly (aged 60 years old or above), patients with chronic diseases, family members and caregivers of infants under six months of age, and pregnant women [6]. Nevertheless, the uptake rate of influenza vaccine among HCWs in China has been reported to remain low as 13.5% in 2015 [7], much lower than that in the United States (78.6%) [8], the United Kingdom (50.4%) [9], and Spain (50.7%) [10]. Provincial influenza vaccination uptake rates differed widely in China partly due to the differential subsidy policies across regions [11]. In 2018, the National Health Committee in China proposed that healthcare facilities should provide influenza vaccination for medical staff for free [12]. This is the first national-wide policy initiative to vaccinate medical practitioners, but it has not been fully implemented across the country. Additionally, HCWs are the major information source about influenza and influenza vaccine for high-risk patients, and the attitudes and beliefs of HCWs can affect the vaccination behaviors of patients, especially children and the elderly [13,14]. Therefore, HCWs play an important role in vaccine promotion, and the vaccine recommendation from vaccinated HCWs to patients had profound influence on improving public health. Previous studies have shown than nearly 40–60% of HCWs in China were not willing to recommend the seasonal influenza vaccine to their patients, which is a large barrier to further improve the vaccine uptake among priority groups [15,16]. 

Public health workers and general practitioners have direct contact with patients, and their recommendations are important for patients’ immunization decisions [17]. However, the medical system and public health system have developed independently in terms of both talent cultivation and staff management in China [18]. Therefore, a long-recognized deficiency of clinical physicians was insufficient education and training on public health [19]. The separation between the two systems also lies in the reimbursement policies, according to which a large proportion of preventive healthcare services, such as influenza vaccination, cannot be reimbursed, while treatment services are usually covered [20]. The lack of effective coordination between the two systems has blocked the integration of medical resources and the share of specialized medical knowledge, which is not conducive to the prevention and treatment of diseases.

In order to increase the coverage and recommendation of influenza vaccination among HCWs, this study used a nationally representative sample to estimate the behaviors of different types of HCWs towards influenza vaccine, aiming to examine whether the separation of public health workers and general practitioners has influence on the seasonal influenza vaccine uptake and recommendation in China.

## 2. Methods 

### 2.1. Study Population

In August to October 2019, a total of 148 community immunization centers from 10 provinces in China were approached to participate in the national survey on influenza vaccination (Strategies of Influenza Vaccination in China study, NCT04038333). In the selected immunization centers, two types of HCWs who were on duty that day were recruited, including public health workers (both doctors and nurses) responsible for vaccination and randomly selected general practitioners. After excluding the samples with missing key demographic variables or outcome variables (*n* = 131), a total of 1159 HCWs, including 627 public health workers and 532 general practitioners, were finally included in the study. This study was approved by Peking University Institutional Review Board (IRB00001052-19076).

**Figure 1:**
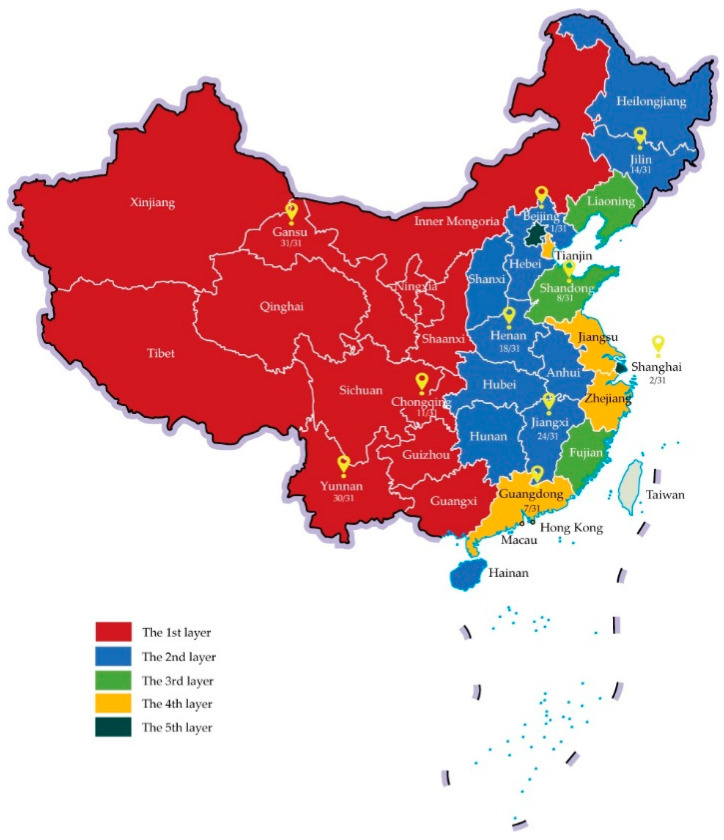
Ten provinces/municipalities/autonomous regions selected in China.

The survey used a multi-stage stratified sampling method. First, 10 provinces/municipalities/autonomous regions were selected based on the Division of Central and Local Financial Governance and Expenditure Responsibilities in the Healthcare Sector released by the General Office of the State Council in 2018, which divided 31 provinces/municipalities/autonomous regions in mainland China into five layers [20]. After considering the location, socioeconomic development, and accessibility, 3, 3, 1, 1, and 2 provinces/municipalities/autonomous regions were chosen from each layer, as showed in Figure 1. Second, in each province/municipality/autonomous region, a capital city (or a well-developed district in municipalities) and a non-capital city (or a less-developed district) were selected. Third, two subdistricts/counties were chosen in each city or district. Fourth, in each subdistrict/county, three or more immunization centers (settled in community health centers or township clinics), and the corresponding neighborhood committees were approached to participate.

### 2.2. Measures

A self-administered and anonymous questionnaire was adopted and modified from the Respiratory Illness and Health Care Worker Study designed by the United States Centers for Disease Control and Prevention [21]. According to the actual conditions in China, we mainly used questions on reasons for not being vaccinated, knowledge of the six target groups for influenza vaccination, and vaccine recommendation behaviors in the present study. The questionnaire can be divided into four parts—(1) demographics; (2) vaccination and recommendation behaviors; (3) knowledge of and attitudes about influenza; and (4) knowledge of and attitudes towards influenza vaccine. Participants chose the extent to which they agree or disagree with scale items based on a five-point Likert scale. In addition, unvaccinated HCWs were asked to answer seven additional questions about the reasons for not being vaccinated. 

### 2.3. Statistical Analysis 

The chi-square test and Mann–Whitney test were used to assess possible differences in socio-demographics, knowledge of and attitudes towards influenza and influenza vaccination among HCWs. Multivariate logistic regression analysis was adopted to predict the influencing factors of vaccination and recommendation behaviors of HCWs. Results were showed as odds ratios (OR) and 95% confidence intervals (CI). A two-sided *p*-value below 0.05 was considered statistically significant. All data were analyzed using STATA, version 13.0 (Stata Corp, College Station, TX, USA).

## 3. Results

### 3.1. Study Sample Characteristics 

Table 1 showed the general characteristics, knowledge, and attitudes about influenza and influenza vaccine among HCWs. The respondents were mainly female (78.4%), and the mean age was 36.4 years old. The overall influenza vaccination rate was 25.3%, and the difference in vaccine uptake between public health doctors (30.5%) and general practitioners (19.2%) was significant (*p* < 0.01). There were significant differences between two kinds of HCWs in terms of age, gender, education level, monthly income, recommendation behaviors for children/the elderly, self-reported severity of influenza, and knowledge on the incubation period for influenza vaccination (all *p* < 0.01). Particularly, 62.4% of public health doctors and 49.1% of general practitioners recommended influenza vaccination to children, and 68.1% of public health doctors and 54.7% of general practitioners recommended it to the elderly. 

### 3.2. Reasons for Not Being Vaccinated

Of the 1159 HCWs in this study, 866 (74.7%) participants were unvaccinated. We further investigated the reasons why they were not vaccinated, including “high vaccine price,” “allergic contraindications,” “worry about adverse reactions,” “low vaccine efficacy,” “vaccine shortage in local areas,” “no need to get vaccinated,” and “inadequate vaccine accessibility” (Table 2). Among the unvaccinated HCWs, “no need to get vaccinated” was the primary reason for both public health workers (205/436) and general practitioners (282/430). High vaccine price was another important reason, as doctors needed to pay for influenza vaccination out of pocket.

### 3.3. HCWs’ Knowledge of Target Groups

Table 3 displayed HCWs’ knowledge of the six target groups for influenza vaccination. It was found that 81.2% general practitioners knew that patients with chronic diseases were one of the priority groups for influenza vaccination, and this proportion was higher than that in public health workers (74.3%, *p* < 0.01). Compared with unvaccinated HCWs, there was a higher proportion of vaccinated HCWs who knew that the target groups for influenza vaccination covered children, the elderly, patients with chronic diseases, HCWs, and family members and caregivers of infants under six months.

### 3.4. Multivariate Predictors of Vaccination

As showed in Table 4, in the multivariate logistic regression model adjusted for socio-demographics, the type of HCWs, knowledge of and attitudes toward influenza and influenza vaccination, and previous history of vaccination, we founded that being public health workers (odds ratios [OR] = 2.20, 95% CI 1.59–3.05, *p* < 0.01), knowing that HCWs are among the priority groups (OR = 2.61, 95% CI 1.63–4.16, *p* < 0.01), self-reporting high severity of influenza (OR = 1.56, 95% CI 1.12–2.17, *p* < 0.01) or high possibility of catching influenza (OR = 1.92, 95% CI 1.36–2.71, *p* < 0.01), and knowing the best timing to vaccinate against influenza (OR = 1.46, 95% CI 1.00–2.12, *p* = 0.05) were positively related to HCWs’ vaccination behaviors.

### 3.5. Multivariate Predictors of Recommendation

In the present study, the recommendation behaviors of HCWs were quantified as whether recommend influenza vaccination to children and the elderly. The first three columns of Table 5 showed the multivariate logistic regression results of recommendation to children. The recommendation behavior was positively associated with vaccination history in the previous season (OR = 2.79, 95% CI 1.99–3.93, *p* < 0.01), being public health workers (OR = 2.10, 95% CI 1.57–2.80, *p* < 0.01), knowing that children are among priority groups (OR = 2.20, 95% CI 1.63–2.96, *p* < 0.01), and knowing the best timing for influenza vaccination (OR = 1.43, 95%CI 1.05–1.95, *p* = 0.02).

Table 5 also showed the results of recommendation to the elderly, indicating that vaccination history in the previous season (OR = 2.80, 95% CI 1.96–4.00, *p* < 0.01), being public health workers (OR = 1.69, 95% CI 1.26–2.25, *p* < 0.01), knowing that the elderly are among priority groups (OR = 3.45, 95% CI 2.13–5.59, *p* < 0.01), knowing the best timing for influenza vaccination (OR = 1.77, 95% CI 1.30–2.42, *p* < 0.01), knowing the incubation period of influenza (OR = 1.47, 95% CI 1.12–1.95, *p* < 0.01) would significantly increase the possibility to recommend influenza vaccination to the elderly.

## 4. Discussion 

To the best of our knowledge, this is the first study using a nationally representative sample to investigate the immunization and recommendation behaviors of influenza vaccine among community-level HCWs in China. This study suggested that the overall coverage rate of influenza vaccine among Chinese HCWs was 25.3%, with public health workers having a higher rate than general practitioners. In addition, the prior vaccination history of HCWs is an important factor affecting their recommendation behaviors.

Despite the strong recommendation for annual influenza vaccination among HCWs made by the WHO and the Chinese Center for Disease Control and Prevention, the uptake rate in practice remains inadequate, which is much lower than that in other countries. Previous studies showed that the knowledge and attitudes of HCWs were positively associated with HCWs’ vaccination behaviors, and their misconceptions about influenza and influenza vaccination may reduce their willingness to get vaccinated [22,23]. Only 26.1% of the HCWs in our study knew that influenza virus in droplets can live for 30 minutes in the air, and only about half of them knew the incubation period of influenza.

The major barrier for both unvaccinated public health workers and general practitioners was “no need to get vaccinated.” The multiple logistic regression model showed that being public health worker, knowing that HCWs are among the priority groups, self-reporting higher severity of influenza or higher possibility of catching influenza, and knowing the best timing to vaccinate against influenza came out to be the independent factors affecting the vaccination status of HCWs, in line with a previous study that showed that the attitudes toward influenza vaccination were the strongest predictor of HCWs’ intention and actual receipt of influenza vaccination [24]. The present study suggested that both public health workers and general practitioners in China should improve their knowledge of and attitudes toward influenza vaccination. Interventions to promote vaccination should consider both the social norm approach and the individual beliefs of targeted HCWs.

It has been reported that lack of doctors’ recommendation was the most common reason for the elderly and children to remain unvaccinated [25,26]. Our study suggested that nearly half of the HCWs have not recommended influenza vaccination to the elderly and children. Prior vaccination history, knowing that elderly/children are among the priority groups, and knowledge on influenza and influenza vaccine (such as the incubation period of influenza and the best vaccination time of influenza vaccine) are important influencing factors of HCWs’ recommendation behaviors. This is consistent with previous studies which showed that vaccinated HCWs were more likely to recommend influenza vaccine to vulnerable patients [9,27,28], and insufficient or misunderstanding knowledge of influenza vaccine was the reason why primary HCWs rarely recommended influenza vaccine to patients [29]. In this sense, measures taken to expand the vaccine coverage among HCWs and widen their knowledge on influenza vaccination could promote their recommendation behaviors, and thus improve the vaccine uptake of patients.

In addition, this study showed that compared with public health workers, general practitioners were less willing to get themselves vaccinated against influenza, and less likely to recommend vaccines to children and the elderly. This may partly explain the low uptake level of influenza vaccine among children and elderly in China. However, in routine practice, after receiving the recommendation from public health workers, most patients would consult the general practitioners or specialists for further consultation, and the advice of the general practitioners or specialists would largely determine whether the patient was finally vaccinated [10,30]. Elderly, child and adults with chronic diseases are the priority group for influenza vaccination and their decision to be vaccine might partly depend on advice received from general practitioners [31]. General practitioners may play an important role in improving the influenza vaccination uptake of community residents. Therefore, the currently separated public health and healthcare services in China’s primary healthcare facilities hinders the progress in vaccine uptake of seasonal influenza.

As China moves to expand primary healthcare services as part of its efforts to achieve universal health coverage, adequate training on general practitioners are imperative. The medical education in China put much emphasis on specialization, resulting in detailed but narrowed knowledge structure of medical practitioners [32]. In Table 3, we can find that vaccination status is highly associated with knowledge of the recommended population instead of the type of HCWs in the primary care facilities. The vaccination rate of general practitioners was significantly lower, indicating a large number of general practitioners lacking basic knowledge of influenza vaccination. Therefore, it is necessary to enhance public health education for general practitioners and integrate public health with clinical medicine in practice. Meanwhile, the responsibility of preventing and controlling infectious diseases is jointly shouldered by public health system and medical system, especially during disease outbreaks such as the COVID-19 pandemic [33]. In the long run, improving influenza vaccination coverage among priority groups needs joint efforts from both public health workers and general practitioners. Furthermore, a recent study showed that in the peak season of influenza, the management of respiratory outbreaks like COVID-19 could be facilitated by expanding influenza vaccination coverage [34]. In this sense, encouraging vaccine uptake and recommendation behaviors among HCWs is helpful in the fight against respiratory outbreaks.

The observational study has a few limitations. First, the vaccination behaviors of HCWs were self-reported, causing potential recall bias despite the low possibility for HCWs to misstate their vaccination status. Meanwhile, self-reported influenza coverage has been reported as a good proxy for recorded vaccination [35]. Second, the participants of this study were public health workers and general practitioners who provide services directly to patients, not medical staff in all departments. Even so, the HCWs enrolled in this study had more contacts with patients from 10 provinces across China, which can sufficiently represent Chinese HCWs who are among the priority groups for influenza vaccination. Third, the data collection was cross-sectional, so causality cannot be inferred with certainty.

## 5. Conclusions

In conclusion, this national cohort study suggested that levels of influenza vaccine uptake and recommendation among HCWs are still relatively low in China, especially among general practitioners. Previous history of vaccination and knowledge of and attitudes toward influenza and influenza vaccine were positively associated with vaccine uptake and recommendation behaviors of HCWs, indicating the importance of adequate and coherent training on immunization for both public health workers and general practitioners. These findings hold promise for the refinement of the current influenza vaccination programs in China.

## Figures and Tables

**Table 1:** Characteristics of surveyed healthcare workers in immunization centers.

	Public Health Workers, *n* (%)	General Practitioners, *n* (%)	*p*-Value
Outcomes			
Vaccination history in the previous season			<0.01 *
Vaccinated	191 (30.5)	102 (19.2)	
Unvaccinated	436 (69.5)	430 (80.8)	
Recommended influenza vaccination to children			<0.01 *
Yes	391 (62.4)	261 (49.1)	
No	236 (37.6)	271 (50.9)	
Recommended influenza vaccination to elderly			<0.01 *
Yes	427 (68.1)	291 (54.7)	
No	200 (31.9)	241 (45.3)	
Knowledge and perception of influenza vaccination			
Self-reported high severity of influenza			0.01
Yes	320 (51.0)	233 (43.8)	
No	307 (49.0)	299 (56.2)	
Self-reported high possibility of catching influenza			0.95
Yes	172 (27.4)	145 (27.3)	
No	455 (72.6)	387 (72.7)	
Knowledge-The best timing to vaccinate is 1–2 months before the influenza epidemic peak			0.18
Yes	491 (78.3)	399 (75.0)	
No	136 (21.7)	133 (25.0)	
Knowledge-The incubation period of influenza is 1–4 days			<0.01 *
Yes	293 (46.7)	292 (54.9)	
No	334 (53.3)	240 (45.1)	
Knowledge-Influenza virus in droplets can live for 30 minutes in the air			0.50
Yes	169 (27.0)	134 (25.2)	
No	458 (73.0)	398 (74.8)	
General characteristics			
Age (years)			<0.01 *
<30	200 (31.9)	103 (19.4)	
30–39	229 (36.5)	205 (38.5)	
40–49	164 (26.2)	160 (30.1)	
≥50	34 (5.4)	64 (12.0)	
Gender			<0.01 *
Female	566 (90.3)	343 (64.5)	
Male	61 (9.7)	189 (35.5)	
Education level			<0.01 *
High school and below	82 (13.1)	69 (13.0)	
Junior college	284 (45.3)	166 (31.2)	
Bachelor and above	261 (41.6)	297 (55.8)	
Monthly income (CNY)			<0.01 *
≤3000	293 (46.7)	147 (27.6)	
3001–4000	114 (18.2)	112 (21.1)	
4001–5000	97 (15.5)	121 (22.7)	
>5000	123 (19.6)	152 (28.6)	
Having children under 6 years			0.96
Yes	236 (37.6)	201 (37.8)	
No	391 (62.4)	331 (62.2)	
Self-reported good health status			0.10
Yes	441 (70.3)	397 (74.6)	
No	186 (29.7)	135 (25.4)	
Having chronic diseases			0.17
Yes	46 (7.3)	51 (9.6)	
No	581 (92.7)	481 (90.4)	
Place of residence			0.74
Rural	238 (38.0)	207 (38.9)	
Urban	389 (62.0)	325 (1.1)	

* *p* < 0.05.

**Table 2:** Reasons for not being vaccinated among healthcare workers (HCWs).

	Public Health Workers (*n* = 436)		General Practitioners (*n* = 430)	
	*n*	%	*n*	%
No need to get vaccinated	205	47.0	282	65.6
High vaccine price	54	12.4	65	15.1
Vaccine shortage in local areas	59	13.5	29	6.7
Worry about adverse reactions	29	6.7	42	9.8
Allergic contraindications	34	7.8	30	7.0
Low vaccine efficacy	22	5.0	39	9.1
Inadequate vaccine accessibility	19	4.4	28	6.5
Other reasons	109	25.0	80	18.6

Note: Reasons for not being vaccinated were not mutually exclusive. The percentage sum of all the reasons were more than 100%, as some HCWs chose more than one reason.

**Table 3:** HCWs’ knowledge of different target groups for influenza vaccination.

	Vaccination Status			Type of HCWs		
	Vaccinated, *n* (%)	Unvaccinated, *n* (%)	*p*-Value	Public Health Workers, *n* (%)	General PractitioNers, *n* (%)	*p*-Value
Knowing HCWs are among target groups	268 (91.5)	615 (77.1)	<0.01 *	504 (80.4)	432 (81.2)	0.72
Knowing children aged 6–59 months are among target groups	232 (79.2)	615 (71.0)	<0.01 *	458 (73.1)	389 (73.1)	0.98
Knowing the elderly ≥60 years are among target groups	282 (96.3)	773 (89.3)	<0.01 *	576 (91.9)	479 (90.0)	0.28
Knowing patients with chronic diseases are among target groups	243 (82.9)	655 (75.6)	0.01 *	466 (74.3)	432 (81.2)	< 0.01 *
Knowing family members and caregivers of infants under six months are among target groups	229 (78.2)	581 (67.1)	<0.01 *	432 (68.9)	378 (71.1)	0.43
Knowing pregnant women are among target groups	124 (42.3)	340 (39.3)	0.36	240 (38.3)	42.1	0.19

* *p* < 0.05.

**Table 4:** Influencing factors of vaccine uptake among HCWs (multiple logistic regression).

Title	OR (95% CI)	*p*-Value
Type of HCWs		
General practitioner	Reference	
public health worker	2.20 (1.59–3.05)	<0.01 *
Know HCWs are among the priority groups for vaccination	2.61 (1.63–4.16)	<0.01 *
Self-reported high severity of influenza	1.56 (1.12-2.17)	<0.01 *
Self-reported high possibility of catching influenza	1.92 (1.36–2.71)	<0.01 *
Know the best timing to vaccinate is 1–2 months before the influenza epidemic peak	1.46 (1.00–2.12)	0.05
Know the incubation period of influenza is 1–4 days	0.74 (0.55–1.00)	0.05
Know influenza virus in droplets can live for 30 minutes in the air	0.86 (0.61–1.21)	0.38
Age (years)		
<30	Reference	
30–39	2.08 (1.35–3.20)	<0.01 *
40–49	2.72 (1.67–4.45)	<0.01 *
≥50	3.50 (1.78–6.91)	<0.01 *
Gender		
Female	Reference	
Male	0.84 (0.56–1.25)	0.38
Education level		
High school and below	Reference	
Junior college	1.24 (0.75–2.04)	0.40
Bachelor and above	1.18 (0.69–2.01)	0.56
Monthly income (CNY)		
≤3000	Reference	
3001–4000	0.95 (0.60–1.49)	0.81
4001–5000	1.13 (0.69–1.85)	0.62
>5000	1.37 (0.77–2.44)	0.28
Having children under 6 years	1.41 (0.99–2.00)	0.06
Self-reported good health status	1.03 (0.74–1.45)	0.85
Having chronic diseases	1.12 (0.65–1.91)	0.69
Place of residence		
Rural	Reference	
Urban	1.01 (0.73–1.40)	0.95

Note: The regression was controlled for province. * *p* < 0.05.

**Table 5:** Influencing factors of HCWs’ vaccination recommendation to children or the elderly (multiple logistic regression).

	Recommendation to Children		Recommendation to the Elderly	
	OR (95% CI)	*p*-Value	OR (95% CI)	*p*-Value
Vaccinated in the previous season	2.79 (1.99–3.93)	<0.01 *	2.80 (1.96–4.00)	<0.01 *
Type of HCWs				
General practitioner	Reference		Reference	
public health worker	2.10 (1.57–2.80)	<0.01 *	1.69 (1.26–2.25)	<0.01 *
Know children (the elderly) are among the target groups for vaccination	2.20 (1.63–2.96)	< 0.01 *	3.45 (2.13–5.59)	<0.01 *
Self-reported high severity of influenza	1.18 (0.88–1.58)	0.26	1.22 (0.91–1.63)	0.19
Self-reported high possibility of catching influenza	1.31 (0.94–1.83)	0.12	1.24 (0.89–1.75)	0.21
Know the best timing to vaccinate against influenza is 1-2 months before the influenza epidemic peak	1.43 (1.05–1.95)	0.02 *	1.77 (1.30–2.42)	<0.01 *
Know the incubation period of influenza is 1-4 days	1.21 (0.92–1.59)	0.18	1.47 (1.12–1.95)	<0.01 *
Know influenza virus in droplets can live for 30 minutes in the air	1.06 (0.79–1.43)	0.70	1.29 (0.95-1.75)	0.10
Age (years)				
<30	Reference			
30–39	1.08 (0.75–1.56)	0.66	0.94 (0.65–1.35)	0.72
40–49	1.18 (0.77–1.8)	0.46	0.95 (0.62–1.47)	0.83
≥50	1.44 (0.77–2.72)	0.26	0.84 (0.45–1.57)	0.58
Gender				
Female	Reference			
Male	1.08 (0.76–1.52)	0.68	0.69 (0.49-0.97)	0.03 *
Education level				
High school and below	Reference			
Junior college	0.59 (0.38–0.92)	0.02 *	1.01 (0.65–1.56)	0.98
Bachelor and above	0.64 (0.4–1.04)	0.07	0.75 (0.47–1.20)	0.23
Monthly income				
≤3000	Reference			
3001-4000	1.63 (1.09–2.43)	0.02 *	1.13 (0.76–1.67)	0.56
4001-5000	1.6 (1.02–2.50)	0.04 *	1.7 (1.08–2.68)	0.02 *
>5000	1.6 (0.95–2.71)	0.08	1.96 (1.15–3.33)	< 0.01 *
Having children under 6 years	1.1 (0.81–1.5)	0.56	0.81 (0.59–1.11)	0.19
Self-reported good health status	1.11 (0.82–1.52)	0.50	1.18 (0.86–1.61)	0.31
Having chronic diseases	1.09 (0.64–1.85)	0.76	1.5 (0.87–2.59)	0.15
Place of residence				
Rural	Reference			
Urban	0.8 (0.6–1.07)	0.12	0.96 (0.72–1.29)	0.78

* *p* < 0.05.
